# Anti-HLA antibodies bound to monocytes altered antibody-mediated platelet phagocytosis and led to mild thrombocytopenia

**DOI:** 10.3389/fimmu.2025.1652134

**Published:** 2025-10-03

**Authors:** Xiuzhang Xu, Nelli Baal, Martin Rick, Dawei Chen, Huaqin Liang, Xin Ye, Wenjie Xia, Hui Ren, Yaori Xu, Yongshui Fu, Gregor Bein, Sentot Santoso

**Affiliations:** ^1^ Institute of Blood Transfusion and Hematology, Guangzhou Blood Center, Guangzhou Medical University, Guangzhou, China; ^2^ Institute for Clinical Immunology, Transfusion Medicine and Hemostasis, Justus-Liebig-University, Giessen, Germany; ^3^ The First School of Clinical Medicine, Southern Medical University, Guangzhou, China; ^4^ Institute of Blood Transfusion and hematology, Guangzhou First People's Hospital, Guangzhou Medical University, Guangzhou, China

**Keywords:** anti-HLA class I antibodies, anti-αIIbβ3 antibodies, platelet clearance, fetal and neonatal alloimmune thrombocytopenia, monocytes

## Abstract

**Background:**

In fetal and neonatal alloimmune thrombocytopenia (FNAIT), maternal antibodies react with alloantigen expressed on fetal platelets, leading to their clearance *via* antibody-dependent phagocytosis. In Caucasians, most FNAIT cases are caused by anti-HPA-1a antibodies. In contrast, anti-HLA class I antibodies are rarely found in FNAIT, but are frequently implicated in cases of platelet transfusion refractoriness (PTR). This phenomenon leads to ongoing debate regarding the role of anti-HLA class I antibodies in FNAIT. In this study, we investigated the platelet clearance mediated by anti-HLA class I antibodies in whole blood both *in vitro* and *in vivo*.

**Methods:**

Clearance of opsonized platelet was analyzed by platelet phagocytosis assay and by antibody administration to Balb/c female mice.

**Results:**

To mimic FNAIT conditions, whole blood was pretreated with anti-HLA antibodies before the phagocytosis of anti-HPA-1a antibody-opsonized platelets. Compared to untreated whole blood, anti-HLA-ABC and anti-HLA-DR IgG antibodies inhibited the phagocytosis of anti-HPA-1a-antibody-opsonized platelets. Similar results were obtained with purified monocytes, indicating that anti-HLA-ABC antibodies bound to monocytes can interfere with antibody-mediated platelet phagocytosis. Furthermore, the administration of anti-MHC-I antibodies to mice led to a significant decrease in the platelet count within 24 h. However, anti-αIIbβ3 antibody administration resulted in significantly higher platelet clearance over different time points. Analysis of antibody-bound platelets showed the presence of anti-αIIbβ3 antibodies on the platelet surface, but not on monocytes. In contrast, anti-MHC-I antibodies were found on both platelets and monocytes. Interestingly, monocytes exhibited higher levels of anti-MHC-I binding than platelets (87.0% vs. 25.5%), most likely because platelets express significantly fewer HLA class I antigens than monocytes, as indicated by our flow cytometric analysis of whole blood.

**Conclusions:**

These results indicated that anti-MHC-I antibodies preferentially bind to monocytes rather than platelets in whole blood and can be cleared by monocytes *via* endocytosis. Furthermore, we found that the presence of anti-HLA class I antibodies did not significantly influence platelet clearance induced by anti-αIIbβ3 antibodies. The question of whether these observations can explain the controversial opinions regarding the relative roles of anti-HLA class I and anti-αIIbβ3 antibodies in FNAIT requires further assessment in a murine model of FNAIT.

## 1 Introduction

Alloantibody-induced platelet clearance is the primary cause of thrombocytopenia and increases bleeding risk in immune-mediated disorders such as platelet transfusion refractoriness (PTR) and fetal and neonatal alloimmune thrombocytopenia (FNAIT) (1–3). Anti-HLA class I antibodies are the most common cause (50%–70%) of PTR (4). Interestingly, while these alloantibodies are frequently found in pregnant women (18%–50%) (5, 6), anti-HLA class I antibody-induced FNAIT is rarely observed (7, 8). In contrast, anti-HPA-1a antibodies, targeting platelet-specific αIIb​β3 ​integrin, are the antibodies most often linked to FNAIT (2). This discrepancy remains largely unexplained, resulting in an ongoing debate about the contribution of anti-HLA class I antibodies to this condition (9, 10).

While numerous case studies have reported an association between maternal alloimmunization against HLA class I antigens and neonatal thrombocytopenia, prospective screening of healthy pregnant women failed to identify an association between the presence of anti-HLA alloantibodies and this condition (9). In contrast, retrospective studies found that anti-HLA class I antibody reactivities were stronger and broader in suspected FNAIT cases than in healthy pregnant controls. Despite this, no association has been detected between anti-HLA antibody strength and the neonatal platelet count (5, 6, 10). However, overall cautious interpretation of the results should be made given to the different time of blood samplings between cases and control cohorts.

In FNAIT, maternal anti-HLA class I antibodies that transfer into the fetal blood circulation encounter HLA class I antigens on both fetal platelets and all fetal nucleated cells. In contrast, in PTR, anti-HLA class I antibodies developed by immunized recipients can only attack HLA class I incompatible platelets from the donors. This phenomenon might explain the difference in the clinical relevance of anti-HLA class I antibodies in FNAIT and PTR.

It is known that HLA class I antigens can be internalized by T-lymphocytes and macrophages/monocytes, but not fibroblasts or B-lymphocytes, through coated pits (11, 12). On monocytes, approximately 30% of HLA class I molecules were found to be internalized within one hour after antibody binding, then transferred to the *trans*-Golgi reticulum and *trans*-Golgi cisternae, suggesting that the process involved receptor recycling (12). A recent, extensive expression atlas of HLA class I molecules derived from proteasome expression data across various body tissues and cell types revealed that platelets express lower levels of HLA class I molecules compared to other immune cells, including monocytes (13). Consequently, it is plausible that anti-HLA class I antibodies preferentially bind to and are internalized by monocytes rather than platelets, thereby evading Fc-dependent antibody-mediated platelet clearance.

Recently, we developed an *in vitro* whole-blood platelet phagocytosis assay (WHOPPA) and demonstrated that platelets opsonized with both anti-HPA-1a and anti-HLA class I antibodies can be engulfed by monocytes—but not by neutrophils—in whole blood *via* FcγRs, a condition that mimics the mechanism of PTR rather than FNAIT (14).

In the current study, we modified our WHOPPA to mimic FNAIT conditions by allowing anti-HLA class I reacted with whole blood cells and confirmed our findings *in vivo* using a murine model. We found that anti-HLA class I antibodies preferentially bind to monocytes in whole blood rather than platelets, thereby blocking platelet clearance and resulting in only mild thrombocytopenia. Moreover, the presence of anti-HLA class I antibodies did not significantly alter platelet clearance induced by platelet-specific anti-αIIbβ3 antibodies. Whether these findings help resolve the ongoing controversy regarding the role of anti-HLA class I antibodies in FNAIT should be further explored using a murine model of FNAIT.

## 2 Materials and methods

### 2.1 Antibodies and sera

Anti-HPA-1a standard sera (03/152) were obtained from the National Institute for Biological Standards and Control (Potters Bar, Hertfordshire, UK). Depending on the stock solution, final dilutions of 1:16 (lot #324735, #331314) or 1:4 (lot #332285) were used. High-titer anti-HLA class I sera were collected from five female donors and characterized in our laboratory. A pool of AB sera was also collected from six healthy male blood donors. This study was conducted in accordance with the declaration of Helsinki and was approved by the Ethics Committee of the Medical Faculty, Justus Liebig University, Giessen, Germany (file no. 82/09 and file no. 05/00).

Mouse monoclonal antibodies (mAbs) against HLA-ABC (clone w6/32; IgG2a), HLA-DR (clone L243; IgG2a), and HLA-DQ (clone Tü169; IgG2a), along with an isotype control (IgG2a), were purchased from Biolegend (Hamburg, Germany). The F(ab′)_2_ IgG fragment was generated by pepsin digestion using the FragIT Z Micro Preparation Kit and purified as recommended by the manufacturer (Genovis, Lund, Sweden). The purity of the F(ab′)_2_ fragment was verified by JESS protein capillary electrophoresis (Bio-Techne, Minneapolis, MN, USA) (**Supplementary Figure 1**). The fluorescently labeled mAbs used for flow cytometry analysis are listed in **Supplementary Table 1**. A mAb against MHC-I (34-1-2S; IgG2a) was purchased from ATCC (VA, USA) and a mAb targeting αIIbβ3 integrin (Leo.F2; IgG2a) was obtained from Emfret Analytics (Shanghai, China).

### 2.2 *In vitro* platelet phagocytosis assay

In this study, platelet phagocytosis by monocytes was performed in the following three different experimental settings: a) untreated platelet-free whole blood, b) antibody-pretreated platelet-free whole blood, and c) isolated monocytes.

a) Phagocytosis of anti-HPA-1a antibody-opsonized platelets by monocytes in untreated platelet-free whole blood was performed using the WHOPPA, as previously described (14). Briefly, 200 µL of pHrodo-labeled platelets (1.5 × 10^7^ platelets) was opsonized with diluted anti-HPA-1a standard sera for 30 min in the dark. After two washes with Dulbecco’s Phosphate Buffered Saline (D-PBS; Anprotec, Bruckberg, Germany) containing 10 mM EDTA and 0.3 mM prostaglandin (Sigma-Aldrich, St. Louis, MI, USA) (PEP, pH 7.4) (10 min, 800 × *g*), platelets were resuspended in 200 µL of Hank’s Balanced Salt Solution (HBSS) containing calcium and magnesium (Anprotec) and 10 mM HEPES (Gibco, Life Technologies, Darmstadt, Germany). Then, 200 µL of pHrodo-labeled, sensitized platelets was mixed with 200 µL of platelet-free whole blood containing 5 × 10^4^ monocytes and incubated for 60 min at 37°C. After erythrocyte lysis, viable monocytes were double-labeled with allophycocyanin (APC)-conjugated anti-CD14 and fluorescein isothiocyanate (FITC)-conjugated anti-CD45 mAbs. These were then counterstained with 1 mM SYTOX Blue (Invitrogen, Paisley, UK) for 30 min in the dark and measured by flow cytometry (FACSCanto II, Becton Dickinson, Franklin Lakes, NJ, USA). The percentage of pHrodo-positive CD45^+^CD14^+^ cells (monocytes that phagocytose opsonized platelets) was analyzed using FlowJo v.10.6.1_CL software (Becton and Dickinson). A minimum of 5,000 CD45^+^CD14^+^ cells were examined. b) Platelet-free whole blood (see above; 200 µL) was treated with mAbs against HLA-ABC, HLA-DR, or HLA-DQ IgG or F(ab′)_2_ fragments (7.5 or 5.0 mg/mL, respectively), or with an anti-HLA serum sample (dilution 1:4 or 1:16) for 30 min. The corresponding isotypes and diluted AB sera served as negative controls. After washing, anti-HPA-1a antibody-opsonized platelets were added and the rate of platelet phagocytosis by monocytes was measured by flow cytometry as described above. c) Monocytes were isolated from whole blood using the MojoSort™ Human Pan Monocyte Isolation Kit (Biolegend, San Diego, CA, USA). The monocyte suspension was adjusted to a concentration of 0.25 × 10^6^/mL in HBSS buffer containing 1 M HEPES. A 200 µL volume of the monocyte suspension (5 × 10^4^ monocytes) was incubated with mAbs against HLA-ABC and HLA-DR. After washing, anti-HPA-1a antibody-opsonized platelets were added, and the rate of platelet phagocytosis by monocytes was measured by flow cytometry as described above.

### 2.3 Flow cytometric analysis of HLA-ABC expression on different blood cells

EDTA-anticoagulated blood (100 µL) was incubated with FITC-labeled anti-HLA-ABC antibody (W6/32; 10 µg/mL) together with BV510-labeled anti-CD45 (HI30; 5 µg/mL), phycoerythrin (PE)-conjugated anti-CD61 (VI-PL2; 3.0 mg/mL), or PB-labeled anti-CD41 (HIPS; 10.0 µg/mL) antibodies for 20 min in the dark following the manufacturer’s protocol. The corresponding fluorescently labeled isotypes served as controls (**Supplementary Table 1**). After spontaneous sedimentation (approximately 20 min), platelet-rich plasma (PRP) was collected and washed with D-PBS (Anprotec) containing 10 mM EDTA and 0.3 µm PGE (Sigma-Aldrich). The remaining platelet-poor blood was subsequently treated with erythrocyte lysis buffer for 5 min and then washed with D-PBS. Fluorescently labeled cells were measured by flow cytometry as described above. Leukocytes were gated *via* the CD45 marker, following which lymphocytes, monocytes, and granulocytes were identified based on forward scatter (FSC) and sideward scatter (SSC) properties. Platelets were identified by the presence of CD41 and CD61 markers. The normalized mean fluorescence intensity (MFI) was calculated as the ratio of the antibody signal to that of the corresponding isotype control.

### 2.4 Platelet clearance caused by anti-MHC I and anti-αIIbβ3 antibodies in mice

MAb 34-1-2S was isolated from Protein-Free Hybridoma Medium (PFHM-II, Life Technologies Corporation, NY, USA) and purified by affinity chromatography as previously described (15). Purified mAb 34-1-2S and purified mAb Leo. F2 (0.25, 1 and 2 mg/kg) were administered to Balb/c (H-2K^d^ haplotype) mice *via* the tail vein. Mouse IgG2a (eBM2a, eBioscience, San Diego, CA, USA) was used as negative control. A small amount of blood (10 µL) was obtained from the tail vein and diluted with 240 µL of buffer for blood cell analysis. Platelet counts were determined using the Veterinary Auto Hematology Analyzer (V-52 series, Mindray, Shenzhen, China) before and at different time points after antibody administration. Furthermore, Balb/c female mice were sequentially injected with mAb 34-1-2S (2 and 0.25 mg/kg), followed 30 min later by Leo.F2 (1 and 0.25 mg/kg). Platelet counts were subsequently measured as described above.

### 2.5 *In vivo* antibody binding to mouse monocytes and platelets

EDTA-anticoagulated blood (50 µL) was absorbed from mice before and after antibody administration (see above). Antibody bound to platelets and monocytes was then quantified by flow cytometry. In brief, PRP was obtained by centrifugation at 100 × *g* for 15 min. Peripheral blood mononuclear cells (PBMCs) were isolated from the remaining whole blood using density gradient centrifugation (1.084; Percoll; GE Healthcare, Uppsala, Sweden). PRP (50 µL; 1 × 10^5^ platelets/µL) in D-PBS (Gibco, Suzhou, China) was stained with FITC-conjugated goat anti-mouse IgG (JacksonImmunoResearch, West Grove, PA, USA) and PE-labeled anti-mouse CD41a antibody (MWReg30, Life Technologies Corp, Carlsbad, CA, USA) at room temperature in the dark. Monocytes were labeled with FITC-conjugated goat anti-mouse IgG (JacksonImmunoResearch) and PE-labeled anti-mouse CD11b antibody (M1/70, Life Technologies Corp). After two D-PBS washes, the cells were resuspended and quantified by flow cytometry (FACS Canto II; BD Biosciences, San Jose, CA, USA).

### 2.6 Statistical Analysis

Data were analyzed using GraphPad Prism Software (Version 10.0.2, GraphPad Software, Inc., La Jolla, CA, USA) and SAS OnDemand for Academics (SAS Institute Inc., Cary, NC, USA). Exploratory analysis was based on descriptive statistics. As the data did not meet the assumptions of parametric tests, non-parametric methods were used. For non-normally distributed percentage data, the Friedman test was applied for group sizes greater than two, while the Wilcoxon test with pairwise comparisons was used for group sizes of two. Power analysis was also performed. To compare data across different time points relative to 0 h, one-way ANOVA was used. For repeated measures involving multiple comparisons, two-way ANOVA was applied.

## 3 Results

### 3.1 Anti-HLA-ABC and anti-HLA-DR antibodies bound to monocytes inhibited anti-HPA-1a-mediated platelet phagocytosis

**Figure 1:**
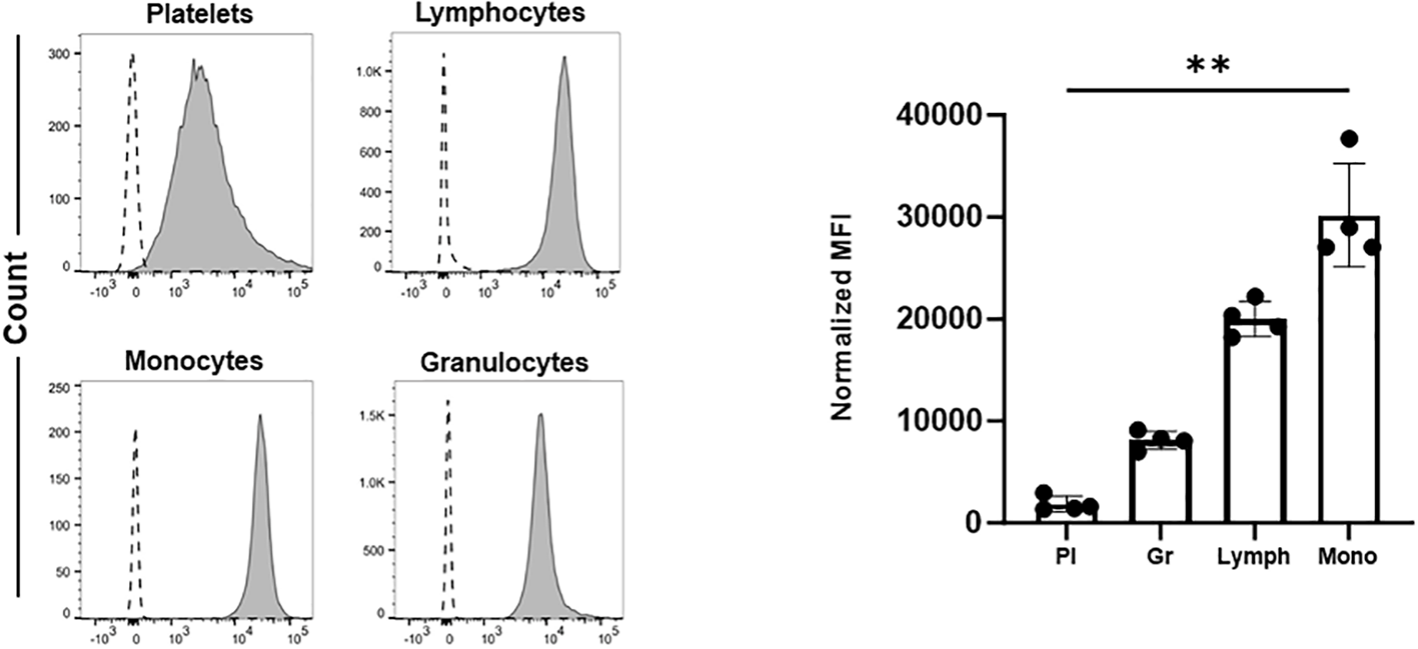
The expression of HLA-ABC on the surface of different blood cells. Platelets (Pl), granulocytes (Gr), lymphocytes (Lymph), and monocytes (Mono) were labeled with FITC-conjugated anti-HLA-ABC antibodies. Cell populations were identified by double labeling using PE-, BV510-, and PB-labeled anti-CD61, anti-CD45, and anti-CD41 antibodies, respectively. Fluorescently labeled cells were quantified by flow cytometry (*see Materials and Methods*). Normalized mean fluorescence intensity (MFI) was calculated as the ratio of the anti-HLA-ABC antibody signal to that of the corresponding isotype control. Statistical analysis was performed by Friedman test. Error bars represent means ± SD. **P≤0.002.

It remains unclear which cells in whole blood are preferentially targeted by anti-HLA-ABC antibodies. To address this, we first used double staining approaches with flow cytometry to measure the binding of anti-HLA-ABC antibodies on different cells in whole blood. As shown in **Figure 1**, HLA-ABC expression on the platelet surface was low compared to that in other blood cells. Notably, monocytes showed the highest HLA-ABC labeling, indicating that anti-HLA-ABC antibodies may preferentially bind to them in whole blood. This result aligns with previous proteasome expression data (13). It is therefore conceivable that anti-HLA-ABC antibodies bound to monocytes can interfere with platelet phagocytosis induced by other platelet-reactive antibodies, such as anti-HPA-1a antibodies.

**Figure 2:**
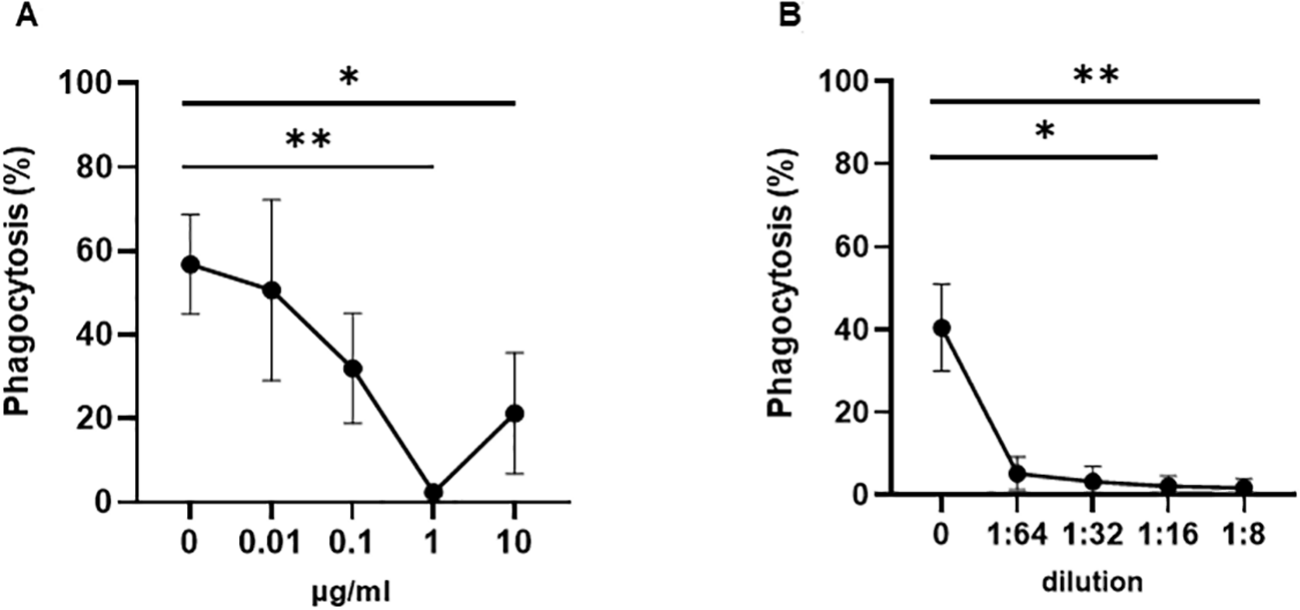
Phagocytosis of anti-HPA-1a antibody-opsonized platelets by whole blood pretreated with anti-HLA class I antibodies. Platelet phagocytosis was performed as previously described (14). **(A)** Platelet-free whole blood was incubated either with mAb W6/32 or **(B)** anti-HLA class I pooled sera at the indicated concentrations. After washing, platelets opsonized with anti-HPA-1a standard serum (dilution 1:16) were added and the percentage of phagocytosis mediated by monocytes pretreated with anti-HLA class I antibodies (% phagocytosis) was determined. Statistical analysis was performed using the Friedman test (one experiment, *n* = 3). Error bars represent means ± SD. *p ≤ 0.03, **p ≤ 0.002.

**Figure 3:**
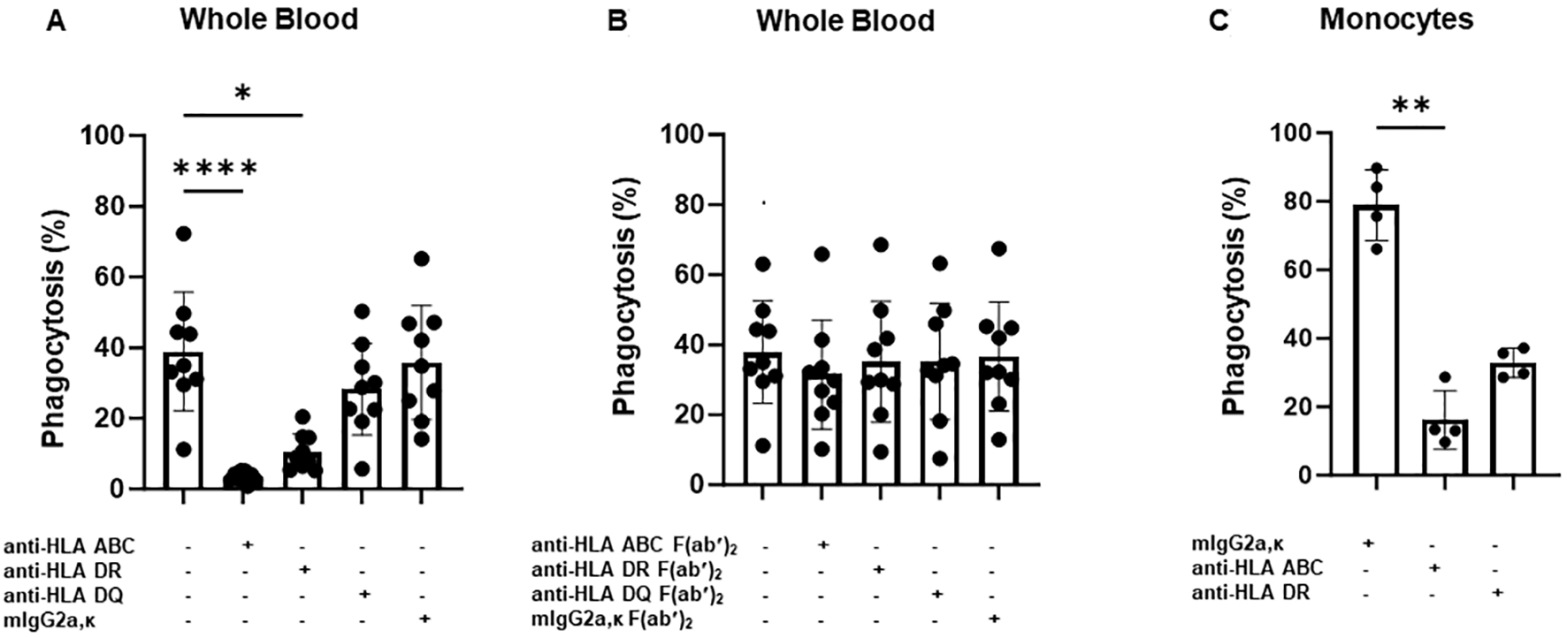
Phagocytosis of anti-HPA-1a antibody-opsonized platelets by whole blood or monocytes derived from different donors in the presence of anti-HLA-ABC, anti-HLA-DR, or anti-HLA-DQ antibodies. PHrodo-labeled platelets were opsonized with anti-HPA-1a standard serum (dilution 1:16) and were then added to platelet-poor whole blood pretreated with anti-HLA-ABC, anti-HLA-DR, anti-HLA-DQ IgG antibodies (7.5 mg/mL), or the F(ab′)_2_ fragment (5 mg/mL) (*n* = 9). Mouse IgG2a and F(ab′)_2_ served as isotype controls. Platelet phagocytosis by monocytes was quantified by flow cytometry as previously described (14) **(A, B)**. **(C)** Platelet phagocytosis performed with isolated monocytes instead of whole blood (*n* = 4). Monocytes were incubated with anti-HLA-ABC or anti-HLA-DR IgG antibodies (7.5 mg/mL) before the phagocytosis of anti-HPA-1a antibody-opsonized platelets. Statistical analysis was performed using the Friedman test. Error bars represent means ± SD. *p ≤ 0.03, **p ≤ 0.002, ****p ≤ 0.0001.

To test this hypothesis, we performed a WHOPPA using whole blood pretreated with anti-HLA-ABC antibody. As shown in **Figure 2**, the pre-incubation of whole blood with mAb W6/32, which targets HLA-ABC, or pooled human sera containing anti-HLA-ABC antibodies, inhibited anti-HPA-1a antibody-opsonized platelet phagocytosis in a concentration-dependent manner. Furthermore, the phagocytosis of anti-HPA-1a antibody-opsonized platelets was inhibited not only by anti-HLA-ABC IgG (mAb W6/32) but also by anti-HLA-DR IgG (mAb L243). However, like the isotype control, the anti-HLA-DQ antibody, mAb Tü169, did not affect platelet phagocytosis (**Figures 3A**). Conversely, pretreatment of whole blood with the F(ab′)_2_ fragment of the anti-HLA-ABC and anti-HLA-DR antibodies did not inhibit the engulfment of anti-HPA-1a antibody-opsonized platelets (**Figure 3B**). To verify that this inhibition was mediated through antibody binding to monocytes, these cells were isolated and subsequently treated with anti-HLA-ABC antibody or anti-HLA-DR IgG as described above (**Figure 3C**). Similar results were obtained, namely, the phagocytosis of anti-HPA-1a antibody-opsonized platelets was inhibited when monocytes were pretreated with anti-HLA-ABC or HLA-DR antibodies. In the control experiment, isotype-treated monocytes could still engulf anti-HPA-1a antibody-opsonized platelets. Although monocytes also express HLA-DQ, the binding of anti-HLA-DQ antibodies did not prevent platelet phagocytosis, unlike that observed with the anti-HLA-DR antibody (**Figure 3A**).

Our results demonstrated that anti-HLA-ABC antibodies not only bound to platelets, triggering their phagocytosis but also strongly interacted with monocytes, consequently inhibiting the phagocytosis of anti-HPA-1a antibody-opsonized platelets. It remains unclear whether a similar mechanism operates *in vivo*.

### 3.2 Anti-MHC-I antibodies induced milder thrombocytopenia than anti-αIIbβ3 antibodies in mice

**Figure 4:**
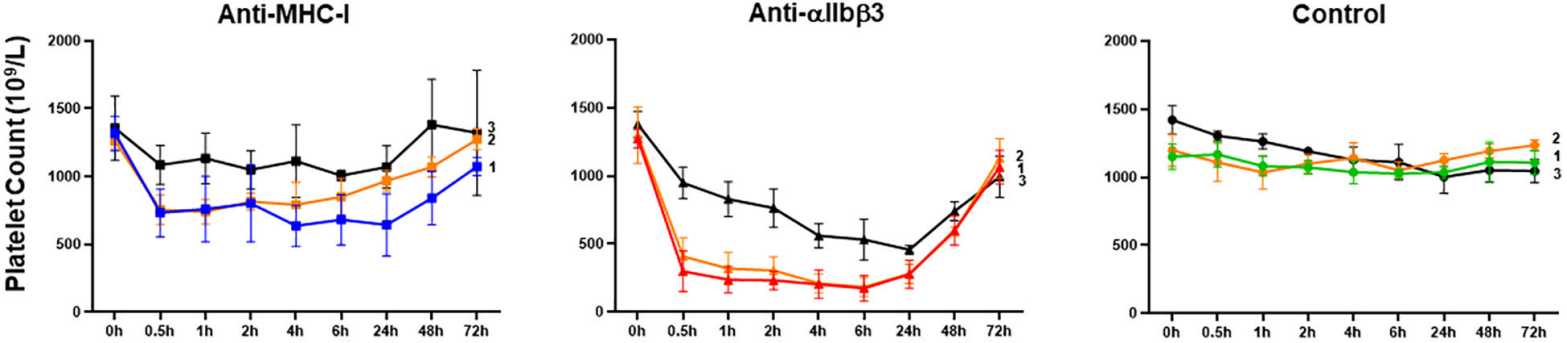
Platelet clearance in mice treated with anti-MHC-I (mAb 34-1-2S) and anti-αIIbβ3 (mAb Leo.F2) antibodies. The antibodies were injected into Balb/c female mice (*n* = 3–5) *via* the tail vein at the doses of 2 (*1*), 1 (*2*), and 0.25 mg/kg (*3*). Mouse IgG2a was injected under the same conditions. The platelet counts, both before and after injection, were determined using an automatic hematology analyzer. The platelet counts at different time points (0.5, 1, 2, 4, 6, 24, 48, and 72 h) were compared against 0 h using one-way ANOVA. Symbols and error bars indicate means ± SD. *P*-values are presented in **Supplementary Table 2**.

To test our hypothesis, we investigated *in vivo* platelet clearance in female Balb/c mice following administration of mAbs against MHC-I (clone 34-1-2S) and αIIbβ3 (clone Leo.F2) (**Figure 4**). The injection of anti-MHC-I antibodies (1 mg/kg) resulted in a significant decrease in the platelet count within 24 h (0 h: 1261.8 ± 59.85 × 10^9^/L *vs*. 755.6 ± 107.93 × 10^9^/L at 0.5 h; 741.0 ± 89.52 × 10^9^/L at 1 h; 815.4 ± 63.28 × 10^9^/L at 2 h; 793.2 ± 168.27 × 10^9^/L at 4 h; 850.6 ± 133.15 × 10^9^/L at 6 h; and 969.8 ± 77.43 × 10^9^/L at 24 h; *p* < 0.05). Platelet counts gradually returned to baseline levels by 72 h post-injection. A similar clearance profile was observed at a higher antibody dose (2 mg/kg). However, the administration of anti-αIIbβ3 antibodies (1 mg/kg) led to significant higher platelet clearance over the different time points (0 h: 1299.8 ± 208.38 × 10^9^/L *vs*. 408.6 ± 137.53 × 10^9^/L at 0.5 h; 319.2 ± 119.30 × 10^9^/L at 1 h; 305.8 ± 100.31 at 2 h; 211.6 ± 70.97 at 4 h; 183.8 ± 69.84 at 6 h; and 280.6 ± 70.77 at 24 h; *p* < 0.01). After 72 h, the platelet counts also returned to a normal value. A comparable result was achieved with a higher antibody dose (2 mg/kg). In the control experiment, the administration of the isotype control did not influence the platelet count over time (*p* > 0.05). When compared with mouse IgG, the platelet counts of Balb/c mice treated with 34-1-2S (1 mg/kg) were significantly decreased over time. However, the platelet counts of Leo.F2-treated mice were continuously very low for 24 h (**Supplementary Table 2**). Next, we sought to define the minimal dose at which anti-MHC-I antibodies could still induce platelet clearance. As shown in **Figure 4**, treatment with 0.25 mg/kg anti-MHC-I antibodies did not induce thrombocytopenia in our mice, as also observed with normal mouse IgG (**Supplementary Table 2**). In contrast, anti-αIIbβ3 antibodies still induced thrombocytopenia at the same dose.

These results indicated that anti-MHC-I and anti-αIIbβ3 antibodies mediate different kinetics of platelet clearance in mice, with anti-MHC-I antibodies inducing mild platelet clearance and anti-αIIbβ3 antibodies promoting strong platelet clearance.

### 3.3 Binding kinetics of anti-MHC-I and Anti-αIIbβ3 antibodies in mice

**Figure 5:**
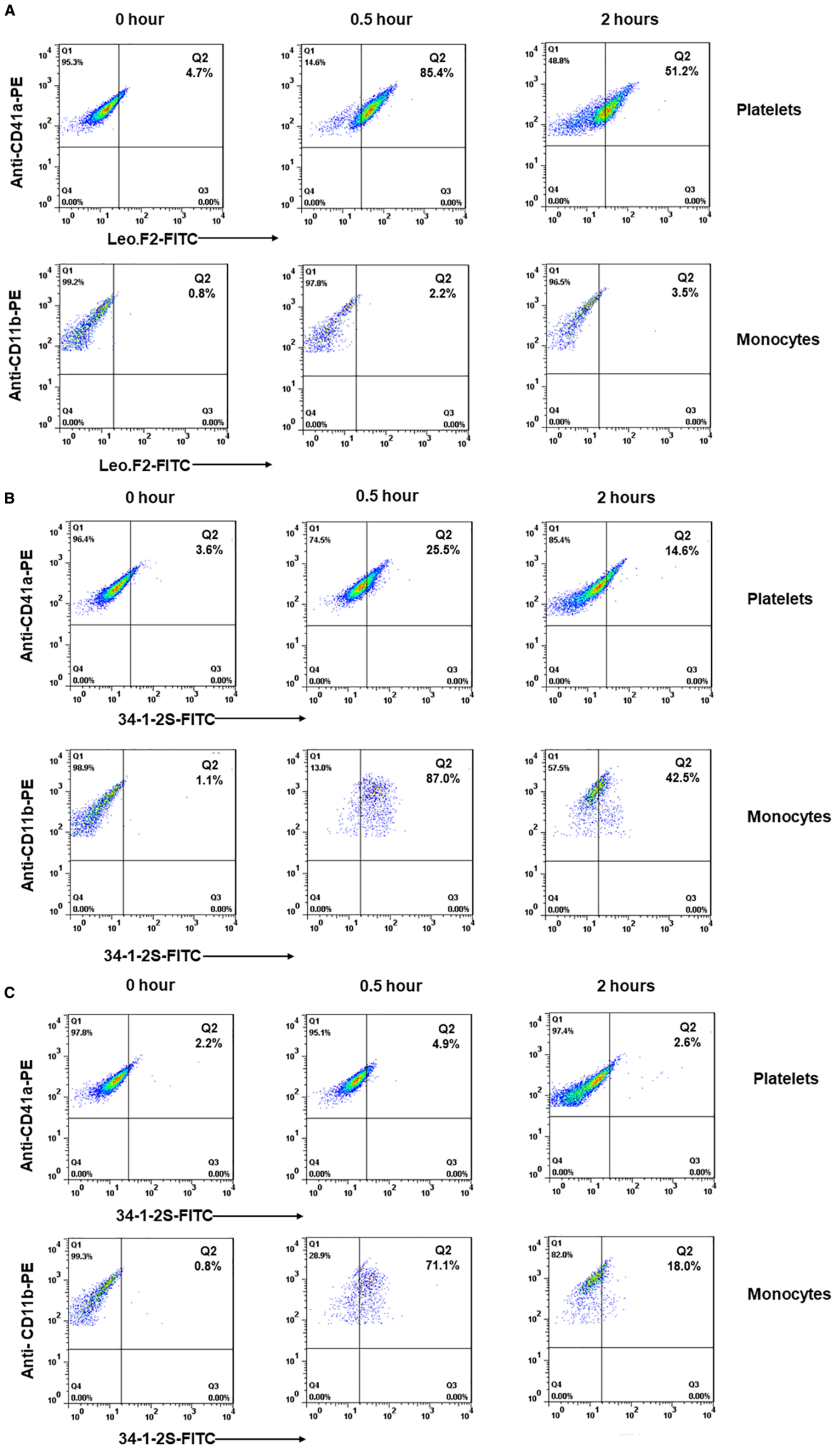
Flow cytometric analysis of the binding of anti-MHC-I and anti-αIIbβ3 antibodies on platelets and monocytes in Balb/c female mice. Platelets and monocytes were isolated from the whole blood before (0 h) and after (0.5 and 2 h) the administration of mAb Leo.F2 (1 mg/kg) **(A)** or 34-1-2S (1 and 0.25 mg/kg) **(B, C)**. Platelets were double-stained with PE-labeled anti-CD41a antibody and FITC-labeled goat anti-mouse IgG. Monocytes were double-stained with PE-labeled anti-CD11b antibody and FITC-labeled goat anti-mouse IgG.

Our *in vitro* results indicated that anti-MHC-I antibodies can bind to monocytes and inhibit the phagocytosis of anti-HPA-1a antibody-opsonized platelets. This phenomenon, however, might not apply under *in vivo* conditions. To examine antibody distribution in whole blood, we isolated platelets and monocytes from Balb/c female mice before and after the administration of anti-MHC-I or anti-αIIbβ3 antibodies (dose 1mg/kg). Bound antibodies were analyzed by flow cytometry using FITC-labeled secondary antibodies. Platelets were identified with PE-labeled anti-CD41a antibodies while monocytes were differentiated with PE-labeled anti-CD11b antibodies (**Supplementary Figure 2**). As shown in **Figure 5A**, strong platelet binding by the anti-αIIbβ3 antibody was found at the 0.5 and 2 h time points. As expected, the binding of platelet-specific anti-αIIbβ3 antibodies on monocytes was not detected (**Figure 5A**). In contrast, strong anti-MHC-I antibody binding to monocytes was evident after 0.5 h, whereas its binding to platelets was significantly weaker (**Figure 5B**). After 2 h, however, monocytes showed heterogeneous staining and some monocytes were negative for MHC-I, indicating antigen membrane mobility triggered by patching, capping, and endocytosis (16). However, at the low dose (0.25 mg/kg), anti-MHC-I antibodies were only detected on monocytes and were not detectable on platelets (**Figure 5C**). In accordance with our earlier observations (**Figure 4**), platelet clearance was not detected at this antibody dose.

These findings suggested that anti-MHC-I antibodies preferentially interact with monocytes rather than platelets *in vivo*, resulting in significantly weaker reactivity compared to the platelet-specific anti-αIIbβ3 antibodies. This may explain the observed lower clearance of anti-MHC-I-opsonized platelets compared to platelet clearance triggered by anti-αIIbβ3 antibodies.

### 3.4 Anti-MHC-I antibody bound to monocytes did not significantly aggravate platelet clearance induced by anti-αIIbβ3 antibodies

**Figure 6:**
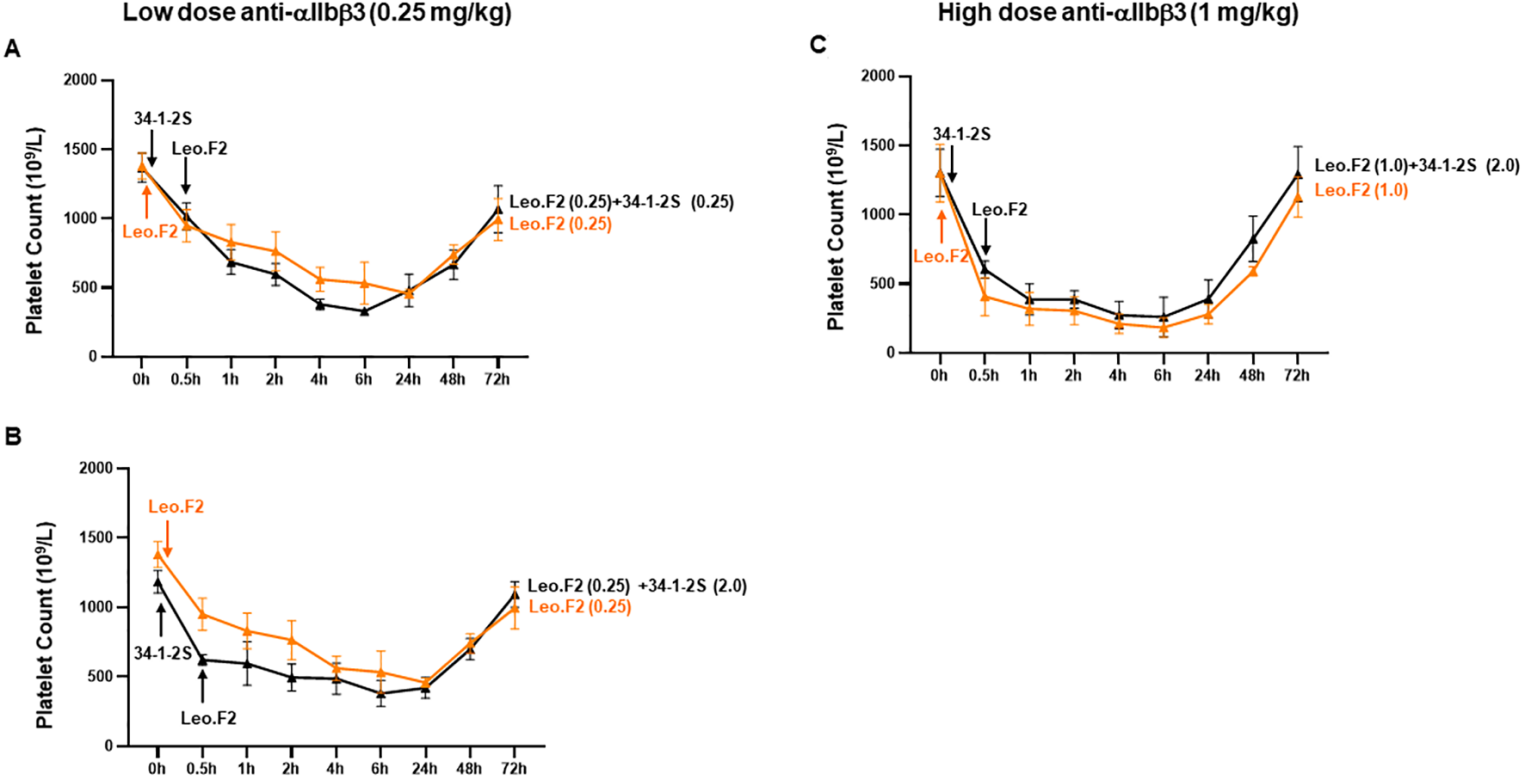
Platelet clearance induced by anti-αIIbβ3 antibodies after the administration of anti-MHC-I antibodies. MAb 34-1-2S (0.25 and 2 mg/kg) was injected first into Balb/c female mice (*n* = 3–4), followed 30 min later by the administration of mAb Leo.F2 (0.25 and 1 mg/kg) antibodies against αIIbβ3. Platelet counts before the injection of 34-1-2S (0 h) and after the administration of Leo.F2 (0.5, 1, 2, 4, 6, 24, 48, and 72 h) were analyzed using an automatic hematology analyzer. Symbols and error bars indicate means ± SD. Comparisons between two groups were undertaken using two-way ANOVA. *P*-values are presented in **Supplementary Table 3**.

Next, we examined whether anti-MHC-I antibodies bound to monocytes could modify anti-αIIbβ3 antibody-induced platelet clearance *in vivo*. At a low anti-αIIbβ3 antibody dose (0.25 mg/kg), co-administration of a low dose of anti-MHC-I antibodies (0.25 mg/kg) did not significantly alter platelet clearance (*p* > 0.05; **Figure 6A**; **Supplementary Table 3**). A similar result was observed when a high dose of anti-MHC-I antibodies (2 mg/kg) was administered 30 min before either the low-dose (0.25 mg/kg) or high-dose (1 mg/kg) anti-αIIbβ3 antibodies (**Figures 6B, C**; **Supplementary Table 3**).

These results suggested that the presence of anti-MHC-I antibodies does not significantly affect the platelet clearance induced by anti-αIIbβ3 antibodies in this murine model.

## 4 Discussion

The conflicting data regarding the role of anti-HLA class I antibodies in FNAIT make it difficult to advise mothers with a history of pregnancy meeting the clinical criteria for FNAIT and in whom only maternal anti-HLA class I antibodies have been detected (10). In this study, we sought to address this concern by analyzing anti-HLA class I antibody-mediated platelet clearance in whole blood both *in vitro* and *in vivo*.

Given that anti-HLA class I antibodies can bind to various cells in whole blood, not just platelets, we modified our WHOPPA protocol. This adaptation involved incubating whole blood first with anti-HLA class I antibodies, followed by incubation with antibody-opsonized platelets. While untreated monocytes readily engulfed anti-HPA-1a-opsonized platelets, monocytes treated with anti-HLA-ABC or anti-HLA-DR IgG antibodies could not. However, this effect disappeared when the F(ab′)_2_ fragments of anti-HLA-ABC and anti-HLA-DR antibodies were employed. In contrast, pretreatment with anti-HLA-DQ antibodies did not inhibit the monocyte-mediated phagocytosis of anti-HPA-1a antibody-opsonized platelets. This effect was most likely attributable to the fact that anti-HLA-DR and anti-DQ antibodies upregulate different mitogen-activated protein kinase (MAPK) signaling pathways (17). Whereas the ligation of HLA-DR molecules on monocytes promoted the phosphorylation of ERK and P38 (inflammatory; IL-1β), that of HLA-DQ induced only P38 phosphorylation (anti-inflammatory; IL-10) (17).

In our previous study, a WHOPPA showed that monocytes, not neutrophils, function as the phagocytes responsible for the engulfment of antibody-opsonized platelets in whole blood (14). In line with this, in the present study, we found that pretreating isolated monocytes with anti-HLA antibodies prevents the phagocytosis of anti-HPA-1a antibody-opsonized platelets. These findings indicate that anti-HLA antibodies bound to monocytes can alter Fc-dependent phagocytosis, thereby inhibiting the engulfment of these opsonized platelets—a phenomenon previously described as “immune phagocytosis inhibition” (18).Given that anti-HLA-DR antibodies also blocked platelet phagocytosis even though platelets lack HLA-DR expression, our observation indicated that anti-HLA antibodies bound to HLA antigens on monocytes (*via* the Fab fragment), along with the crosslinking of FcγRs on monocytes (*via* the Fc fragment), are likely responsible for this inhibitory mechanism. However, whether these mechanisms also apply *in vivo* is currently unclear.

Our *in vivo* study, which involved administering anti-MHC-I antibodies (34-1-2S; 1 mg/kg) to Balb/c female mice, showed that the platelet count was significantly lower in anti-MHC-I antibody-treated mice than in animals receiving the isotype control within 24 h. After 30 min, the platelet count dropped from 1261.8 ± 59.85 × 10^9^/L to 755.6 ± 107.93 × 10^9^/L. However, the platelet counts had normalized by 72 h post-treatment. Notably, the administration of anti-αIIbβ3 antibodies (Leo.F2; 1 mg/kg) resulted in significantly enhanced platelet clearance over different time points relative to that seen with anti-MHC-I antibodies. After 30 min, the platelet count fell to 408.6 ± 137.53 × 10^9^/L. An analysis of antibody-bound platelets (after 0.5 and 2 h) showed the presence of anti-αIIbβ3 antibodies on the surface of platelets, but not that of monocytes, as expected for platelet-specific antibodies. In contrast, anti-MHC-I antibodies were found on both platelets and monocytes. Interestingly, monocytes displayed higher levels of anti-MHC-I binding than platelets (87.0% *vs*. 25.5% and 42.5% *vs*. 14.6% after 30 min and 2 h, respectively), most likely because platelets express significantly lower levels of HLA class I antigens compared to monocytes, as shown by our flow cytometric analysis with whole blood and other approaches (13). Furthermore, after 2 h, some monocytes became negative for MHC-I, indicative of antigen endocytosis induced by anti-MHC-I antibodies (12, 16). These results indicate that anti-MHC-I antibodies preferentially bind to monocytes rather than platelets and can be cleared by monocytes *via* endocytosis. This may explain the observed lower clearance by anti-HLA class I antibody-opsonized platelets compared to platelet clearance triggered by anti-αIIbβ3 antibodies.

In addition, we found that pretreatment with anti-MHC-I antibodies before the administration of anti-αIIbβ3 antibodies reduced platelet clearance. However, this difference was not significant compared to mice receiving anti-αIIbβ3 antibodies alone. This contrasts with our *in vitro* findings, indicating that targeting monocytes and platelets separately does not fully mimic *in vivo* conditions. Nevertheless, our *in vivo* results are in accordance with clinical observations. In FNAIT cases caused by anti-HPA-1a antibodies, no association was found between the presence of anti-HLA class I antibodies and the neonatal platelet count, birth weight, or the occurrence of intracranial hemorrhage (9).

**Figure 7:**
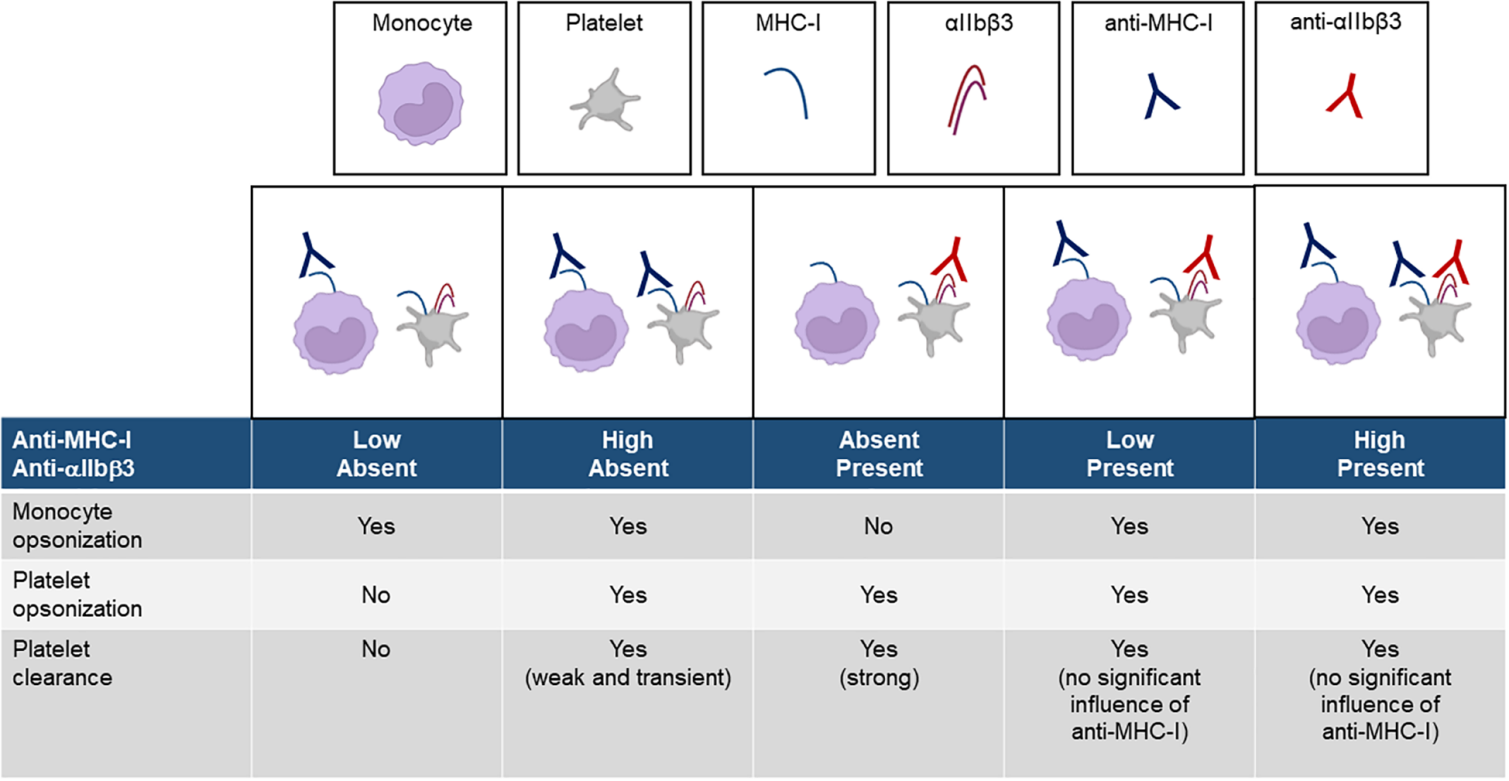
Schematic of the platelet clearance induced by anti-MHC-I and anti-αIIbβ3 antibodies.

The different conditions that likely occur *in vivo* are illustrated in **Figure 7**. A low dose of anti-MHC-I antibodies does not induce platelet clearance, while a high dose produces only mild thrombocytopenia. In contrast, anti-αIIbβ3 antibodies elicit markedly stronger platelet clearance at both low and high doses. The coexistence of anti-MHC-I antibodies, regardless of dose, does not significantly impair the severity of thrombocytopenia induced by anti-αIIbβ3 antibodies.

Recently, other platelet clearance mechanisms besides Fc-dependent phagocytosis have been described in cases of PTR. These include platelet activation, platelet desialylation, and intrinsic features such as IgG subclasses, IgG glycosylation (relating to its ability to activate complement), and antigen specificity (19–21). Some of these parameters could also be relevant to the mechanism of FNAIT and may explain the clinical diversity of anti-HLA class I antibody-mediated FNAIT.

Nevertheless, unlike PTR conditions, we showed in this study that anti-HLA class I antibodies not only react with platelets but also interact with monocytes in whole blood, thereby inhibiting antibody-mediated platelet clearance. This additional mechanism induced milder thrombocytopenia than that observed with platelet-specific anti-αIIbβ3 antibodies. Whether these findings can explain the controversial opinions regarding the role of anti-HLA class I antibodies in FNAIT remains unclear and needs to be further addressed in a murine model of FNAIT.

## Data Availability

The original contributions presented in the study are included in the article/**Supplementary Files**, further inquiries can be directed to the corresponding author/s.
