# Supplementary material for: Anti-HLA antibodies bound to monocytes altered antibody-mediated platelet phagocytosis and led to mild thrombocytopenia
Source: Front Immunol. 2025 Oct 3;16:1652134. doi: 10.3389/fimmu.2025.1652134 (PMC12531028; doi:10.3389/fimmu.2025.1652134)
Supplement: Supplementary file 1 [file DataSheet1.pdf]

Supplementary Figure 1

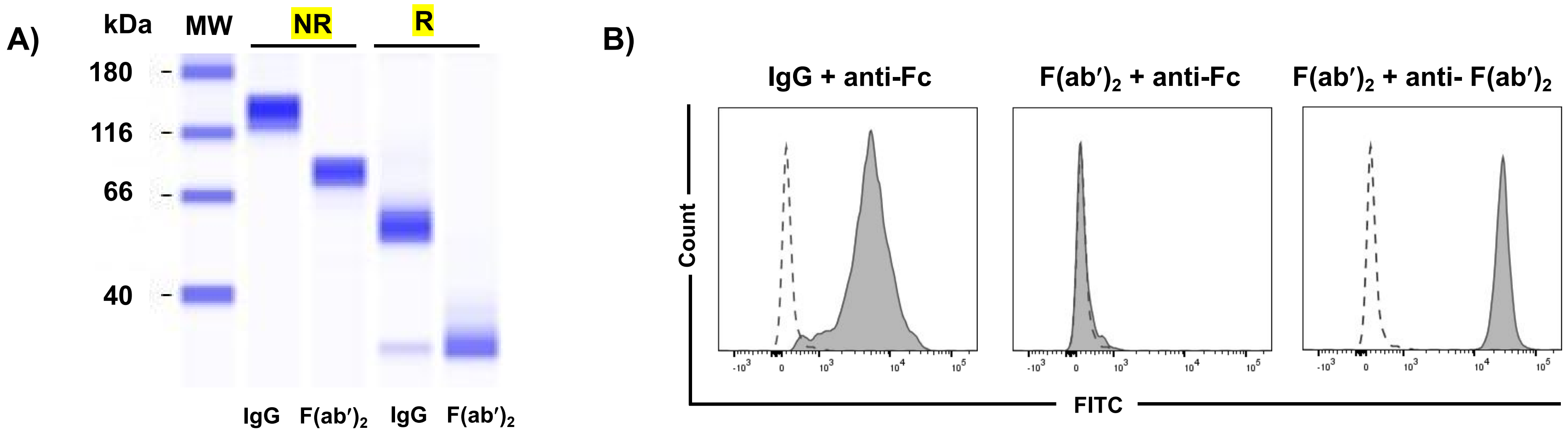

Supplementary Figure 2

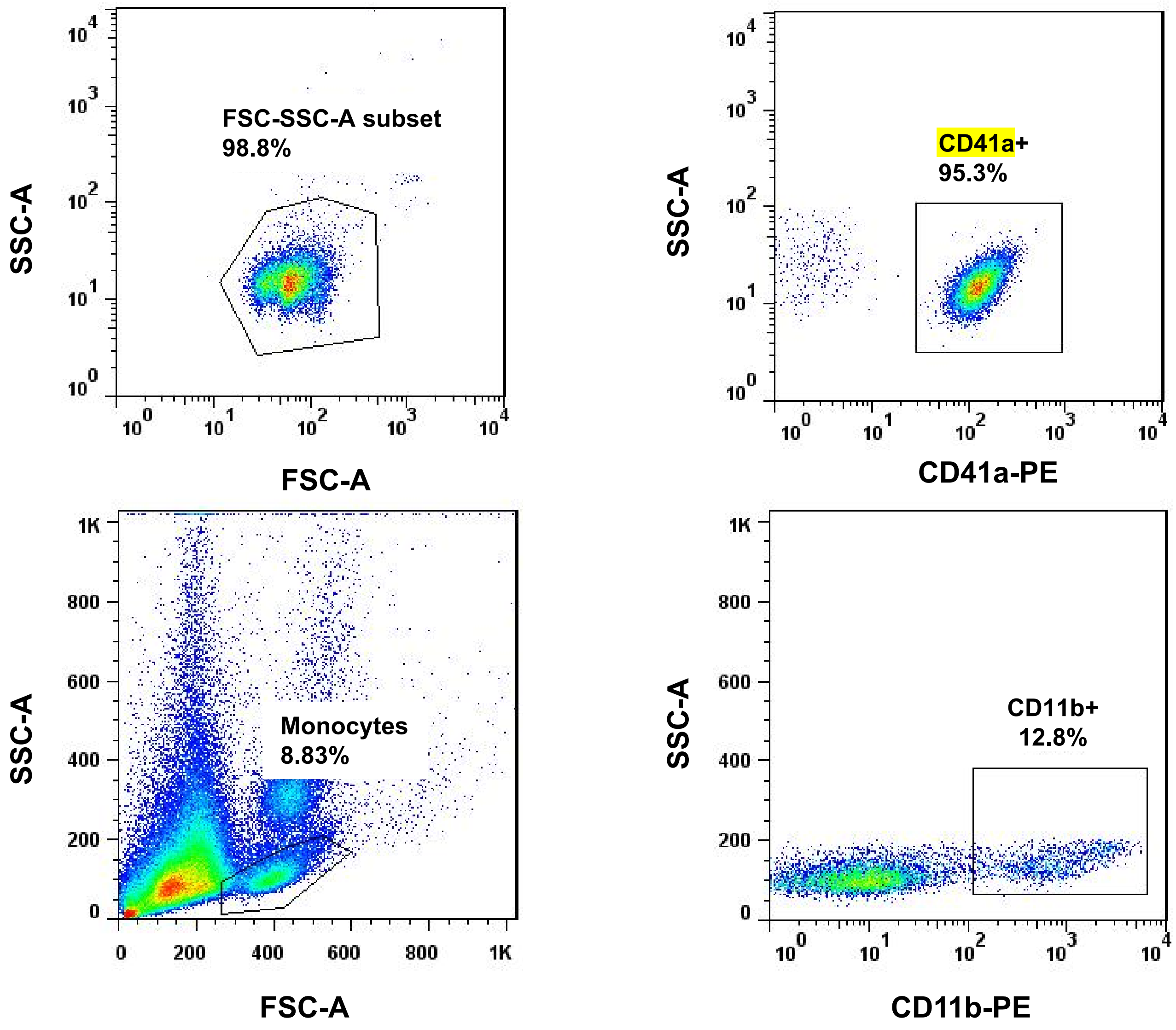

Supplementary Table 1

Number	Antibodies	Clone	Fluorochrom	Manufacturer	IgG Subclasses	Stock Conc. (mg/mL)	Final Conc. (µg/mL)
1	CD14	M5E2	APC	BioLegend	IgG2aκ	0.2	10.0
2	CD45	2D1	FITC	BioLegend	IgG1κ	0.1	4.0
3	HLA-ABC	W6/32	FITC	BioLegend	IgG2aκ	0.2	10.0
4	CD61	VI-PL2	PE	BioLegend	IgG1κ	0.06	3.0
5	CD45	HI30	BV510	BioLegend	IgG1κ	0.1	5.0
6	CD41	HIPS	PB	BioLegend	IgG1κ	0.2	10.0
7	Rabbit-anti-human IgG F(ab′)$_2$	-	FITC	Dako	IgG	0.87	6.5
8	Rabbit-anti-mouse IgG F(ab′)$_2$	-	Alexa Fluor® 488	Jackson	IgG1κ	1.5	11.25
9	Rabbit-anti-mouse IgG F(ab′)$_2$ specific	-	Alexa Fluor® 488	Jackson	IgG	1.5	11.25
10	Mouse IgG	MOPC-21	APC	BioLegend	IgG1κ	0.2	10.0
11	Mouse IgG	MOPC-21	PE	BioLegend	IgG1κ	0.2	10.0
12	Mouse IgG	MOPC-21	PB	BioLegend	IgG1κ	0.2	10.0
13	MHC-I	34-1-2S	-	ATCC	IgG2aκ	4.5	-
14	αIIbβ3	Leo.F2	-	Emfret	IgG2aκ	0.5	-
15	HLA-DR	L243	-	BioLegend	IgG2aκ	0.2	10
16	HLA-DQ	Tü169	-	BioLegend	IgG2aκ	0.2	10
17	CD11b	M1/70	PE	Invitrogen	IgG2bκ	0.2	4.0

Supplementary Table 2

Antibody Dose (mg/kg)	Platelet Count (Mean ± SD)								
	0h	0.5h	1h	2h	4h	6h	24h	48h	72h
34-1-2S (1.0)	1261.80±59.85	755.60±107.93 *P*=0.0017	741.00±89.52 *P*=0.001	815.40±63.28 *P*=0.0003	793.20±168.27 *P*=0.0111	850.60±133.15 *P*=0.0082	969.80±77.43 *P*<0.0001	1070.00±73.62 *P*=0.0173	1274.60±77.14 *P*=0.9921
34-1-2S (2.0)	1318.00±126.33	734.40±177.52 *P*=0.0034	761.60±242.62 *P*=0.0164	802.60±283.11 *P*=0.0362	637.20±149.54 *P*=0.0003	682.00±185.57 *P*=0.0023	644.60±228.35 *P*=0.007	841.40±196.98 *P*=0.0282	1073.80±66.98 *P*=0.0597
34-1-2S (0.25)	1357.00±236.41	1085.00±144.45 *P*=0.1264	1133.75±187.92 *P*=0.1154	1051.25±141.22 *P*=0.1000	1113.50±269.55 *P*=0.2903	1008.25±38.50 *P*=0.1654	1070.00±158.12 *P*=0.6186	1382.25±334.98 *P*=0.9999	1323.25±462.22 *P*=0.9999
Leo.F2 (1.0)	1299.80±208.38	408.60±137.53 *P*=0.002	319.20±119.30 *P*=0.0016	305.80±100.31 *P*=0.0003	211.60±70.97 *P*=0.0004	183.80±69.84 *P*=0.0004	280.60±70.77 *P*=0.0011	590.40±33.56 *P*=0.007	1128.40±145.85 *P*=0.5315
Leo.F2 (2.0)	1272.0±67.77	298.40±148.38 *P*=0.0008	237.60±96.74 *P*=0.0002	232.60±67.99 *P*<0.0001	204.00±103.90 *P*=0.0001	174.80±93.14 *P*<0.0001	277.60±104.09 *P*=0.0003	601.20±106.46 *P*=0.0017	1064.00±123.85 *P*=0.0789
Leo.F2 (0.25)	1379.00±93.36	949.00±115.05 *P*=0.0314	828.67±128.15 *P*=0.0078	763.67±140.78 *P*=0.0192	560.33±86.94 *P*=0.0018	533.00±151.09 *P*=0.0272	456.67±32.02 *P*=0.0058	740.00±69.42 *P*=0.0181	993.67±151.93 *P*=0.1716
mIgG (1.0)	1197.00±119.08	1109.20±139.72 *P*=0.279	1036.00±121.44 *P*=0.3059	1099.20±68.47 *P*=0.6194	1140.60±113.54 *P*=0.9442	1050.80±59.64 *P*=0.2679	1123.40±50.96 *P*=0.7208	1192.00±50.98 *P*=0.9998	1235.40±37.82 *P*=0.9605
mIgG (2.0)	1151.60±94.20	1167.20±91.65 *P*=0.9997	1083.20±71.86 *P*=0.798	1072.00±50.97 *P*=0.6981	1036.80±86.52 *P*=0.3211	1026.80±30.09 *P*=0.2693	1036.40±46.28 *P*=0.2477	1111.80±145.85 *P*=0.9777	1108.00±87.91 *P*=0.9698
mIgG (0.25)	1420.75±104.87	1304.25±34.93 *P*=0.3194	1262.75±55.78 *P*=0.2635	1191.25±14.24 *P*=0.0993	1127.25±92.83 *P*=0.1367	1110.50±129.78 *P*=0.0669	1001.75±121.82 *P*=0.0706	1051.75±91.14 *P*=0.0827	1046.25±86.20 *P*=0.0812

Supplementary Table 3

Antibody Dose (mg/kg)	Platelet Count (Mean ± SD)								
	0h	0.5h	1h	2h	4h	6h	24h	48h	72h
Leo.F2 (0.25)	1379.00±93.36	949.00±115.05	828.67±128.15	763.67±140.78	560.33±86.94	533.00±151.09	456.67±32.02	740.00±69.42	993.67±151.93
Leo.F2 (0.25)+34-1-2S (0.25)	1368.00±104.31<br>*P*>0.9999	1016.33±97.75<br>*P*=0.9974	686.00±89.02<br>*P*=0.8611	597.00±79.00<br>*P*=0.8078	380.33±39.40<br>*P*=0.3820	330.67±17.50<br>*P*=0.7544	481.00±117.55<br>*P*>0.9999	667.33±105.88<br>*P*=0.9874	1068.33±168.59<br>*P*=0.9997
Leo.F2 (0.25)+34-1-2S (2.0)	1182.00±80.72<br>*P*=0.3803	638.33±66.49<br>*P*=0.1961	594.33±156.16<br>*P*=0.6756	494.00±97.55<br>*P*=0.4255	486.00±112.15<br>*P*=0.9924	379.00±93.72<br>*P*=0.8949	419.67±74.81<br>*P*=0.9978	698.00±75.43<br>*P*=0.9986	1092.00±92.37<br>*P*=0.9903
Leo.F2 (1.0)	1299.80±208.38	408.60±137.53	319.20±119.30	305.80±100.31	211.60±70.97	183.80±69.84	280.60±70.77	590.40±33.56	1128.40±145.85
Leo.F2 (1.0)+34-1-2S (2.0)	1302.50±170.05<br>*P*>0.9999	603.50±62.93<br>*P*=0.4065	387.75±113.34<br>*P*=0.9912	387.25±63.32<br>*P*=0.8380	274.50±99.75<br>*P*=0.9741	260.00±144.12<br>*P*=0.9875	391.00±136.83<br>*P*=0.8835	825.25±164.47<br>*P*=0.4405	1292.25±198.18<br>*P*=0.8951
